# Teaching global health with simulations and case discussions in a medical student selective

**DOI:** 10.1186/s12992-015-0111-2

**Published:** 2015-07-04

**Authors:** Nathan S Bertelsen, Michelle DallaPiazza, Mary Ann Hopkins, Gbenga Ogedegbe

**Affiliations:** Departments of Medicine and Population Health, New York University School of Medicine, Bellevue Hospital Center, 16N1, 462 First Ave, New York, NY 10016 USA; Koç University School of Medicine, Rumeli Feneri Campus, Sariyer / Istanbul, Turkey; Department of Medicine, Division of Infectious Diseases, New York University School of Medicine, New York, NY USA; Department of Surgery, Director of Global Health Initiatives, New York University School of Medicine, New York, NY USA; Departments of Population Health and of Medicine, New York University School of Medicine, New York, NY USA; Global Institute of Public Health, New York University, New York, NY USA

**Keywords:** Medical education, Global health, International health, Cross-cultural sensitivity, Cultural competence, Health disparities, Tropical medicine, Non-communicable diseases, Simulation education, Ethics, Health and human rights

## Abstract

**Background:**

Among US medical schools, demand for Global Health (GH) programs continues to grow. At the same time, cultural competency training has become a priority for medical students who will care for an increasingly diverse US patient population. We describe a pilot period for a new GH Selective designed to introduce medical students to global medicine and enhance culturally-sensitive communication skills.

**Methods:**

As a 4-week clinical clerkship, the GH Selective was offered annually over a three-year period to a total of 33 students. Activities included clinical assignments, cultural competency and clinical skills simulations, patient case discussions in tropical medicine, journal clubs, and lectures. Faculty assessments of student performance and student evaluations of course content were focused on 6 course objectives, adapted from standardized GH objectives.

**Results:**

For each offering of the GH Selective, at least 40 faculty members and fellows volunteered over 200 teaching hours from 11 medical school departments. Student feedback was consistently positive through competency-based curricular evaluations. As a result of its successes, the course is now offered on a biannual basis.

**Discussion:**

Experiential, student-centered teaching employed in this course proved successful as an introduction to delivery of evidence-based and culturally sensitive GH. Special emphasis on working with standardized patients in interdisciplinary and cross-cultural simulations provided students with clinical skills applicable for care provided both locally and on international rotations.

**Conclusion:**

With a special emphasis on cross-cultural sensitivity, this pilot elective trained future practitioners in fund of knowledge, clinical skills, and service delivery methods in GH.

**Electronic supplementary material:**

The online version of this article (doi:10.1186/s12992-015-0111-2) contains supplementary material, which is available to authorized users.

## Background

The growing field of Global Health (GH) spans every scientific, clinical, and social science discipline, and skills developed in the study of GH are relevant to all health professions and specialties [1]. Furthermore, with an increasingly mobile global population [2], today’s US physicians require training in the global burden of disease and health disparities in order to care for a diverse patient population [3].

As a result, the demand for GH curricula and cultural competency training in medical schools and residencies has increased over the past decade [4], and the Association of American Medical Colleges (AAMC) has identified cultural competency as a universal educational priority [5]. In response to this growing need, institutions across the US have developed GH programs that highlight a number of important educational priorities: fund of knowledge on the global burden of disease, clinical skills in resource-limited settings, and cultural competence with emphasis on the economic and social determinants of health-related behaviors [6–9].

In addition to enhancement of cultural competence [10], GH educational programs have been shown to have a broader application in improving physical exam skills [11] and responsible use of routine diagnostic measures and expensive resources [12]. Longitudinal medical education programs in GH ensure training that is culturally sensitive, multidisciplinary, and committed to equitable and sustainable collaboration with global partners [13–15].

In this narrative review, we report our methods and evaluations for developing a four-week, multi-disciplinary GH Selective, designed as an introduction to a broader GH program within New York University (NYU) School of Medicine, over a 3-year pilot period. With a special emphasis on cross-cultural sensitivity, the primary aims of the course are to introduce future practitioners to fund of knowledge, clinical skills, and service delivery methods relevant to both global and to culturally-diverse domestic settings, and to prepare students for international clinical rotations or research projects. To meet these goals, we use a variety of teaching methods and educational settings, including simulated experiential learning exercises.

## Methods

We designed the GH Selective to be integrated into larger educational objectives at NYU School of Medicine: the Curriculum for the 21 Century (C21). Novel components of the C21 emphasize personalization, interdisciplinary curricular elements, and professional development [16]. Among various options, which include GH, all NYU medical students are required to choose and complete a 12- week “concentration” suited to their research and/or clinical interests. In C21, a “selective” is a clinical block/clerkship that is as rigorous as a required block, and students complete a required number of selectives by selecting from various options, which include GH, the Cardiac System, Emergency Medicine, Gender and Health, and many others. As pre-requisite to the GH concentration, the GH Selective was designed with five core activities: clinical assignments at Bellevue Hospital Center (BHC), a municipal academic teaching hospital in New York City; cultural competency and clinical skills simulations; patient case discussions in tropical medicine; literature review and journal clubs; and lectures. Shared learning objectives across all activities for the course were adapted from standardized GH objectives [17] (Table 1).

**Table 1:** Shared learning objectives/GH competencies

Develop and practice cross-cultural communication skills
Appreciate cultural and social determinants of health-related needs and behavior
Deepen fund of knowledge of global health disparities and tropical diseases
Build competencies to prepare for clinical services and/or research in resource-limited settings
Understand ethical issues in working with underserved populations
Use leadership principles and skills to improve health care delivery at the population level both abroad and at home

As a four-week clinical block, the GH Selective initially was offered annually over a three-year pilot period. To date, the selective was completed by 9 medical students in 2012, 12 medical students in 2013 and 12 medical students in 2014. Most students were in their third year of medical school. There were many learning activity components and sessions in this selective: each student participated in at least 2 half-days per week in related clinical assignments, 10 cultural competency and clinical skills simulations, 1 microbiology workshop, 14 patient case discussions, 3 journal club sessions, and 8 lectures/conferences. These components are arranged in a general weekly schedule in Table 2, and are described as follows.

**Table 2:** General weekly schedule

Monday	Tuesday	Wednesday	Thursday	Friday
8:00 - 12:00	8:00 - 12:00	8:00 - 12:00	8:00 - 12:00	8:00 - 12:00
Clinic assignments / Independent study / Lecture / conference	Clinic assignments / Independent study / Lecture / conference	Clinic assignments / Independent study / Lecture / conference	Clinic assignments / Independent study / Lecture / conference	Clinic assignments / Independent study / Lecture / conference
12:00 - 1:00 Lunch	12:00 – 1:00 Lunch	12:00 - 1:00 Lunch	12:00 - 1:00 Lunch	12:00 - 1:00 Lunch
Clinical skills simulations	1:00 - 2:30	1:00 - 2:30	1:00 - 2:30	Clinical skills simulations
	Case discussions	Case discussions	Case discussions (or journal club)	
	3:00 - 4:30	3:00 - 4:30	3:00 - 4:30	Microbiology workshop
	Case discussions	Case discussions / Clinical skills simulations	Case discussions (or journal club)	
Clinical assignments / Independent study	Evenings	Evenings	Evenings	Evenings

### Clinical assignments

We assigned students to clinics at BHC, an 828-bed municipal teaching hospital and referral center in New York City with a large ambulatory care center that sees over 500,000 patients from across the exceptionally diverse population of the city. According to the 2012 United States Census, 37 % of 8.3 million total residents of New York City were born outside of the United States. Forty-nine percent of foreign-born and six percent of native-born residents speak a language other than English at home [18]. This high level of diversity offered students the opportunity to work with patients from a wide variety of cultures, languages and countries of origin during their clinical assignments.

At BHC, students worked directly with a faculty member in one of 5 clinics: Adult Infectious Diseases (ID), Adult HIV Primary Care, Pediatric ID, Leprosy, and the Bellevue/NYU Program for Survivors of Torture. In addition, each student participated on inpatient rounds with an ID faculty member or fellow at least once during the four-week elective block. At the end of the course, the students submitted a clinical case write-up that included both a proposed pathogenesis diagram and a literature-based discussion.

### Cultural competency and clinical skills simulations

Experiential learning simulations for the GH selective used a multi-disciplinary approach to introduce the students to pertinent patient-centered, cross-cultural communication and clinical skills for resource-limited settings, across several different clinical cases and disciplines. Students were placed in the simulated position of clinicians who need to evaluate and manage both live standardized patients (actors) and simulated patients (high-technology mannequins), who presented with a wide variety of health-related needs. Common themes included tropical medicine, non-communicable diseases, public health campaigns, healthcare delivery systems and triage, ethical issues, human rights topics, and health care disparities.

The simulations were hosted by the New York Simulation Center for the Health Sciences (NYSIM). As one of the nation’s largest urban health science training facilities, NYSIM provided the space, technology, and guidance for simulated patient encounters. The scenarios were typically set overseas and based on field experience of the facilitating faculty (Table 3).

**Table 3:** Clinical skills simulations (“standardized patients” are live actors playing the role; “simulated patients” are high-technology mannequins that are operated by faculty)

Clinical theme	Exercise	Simulation method	Simulated site	Selected activity objectives
Emergency care/Trauma response	Obstetrical emergencies in a rural primary health care facility	Standardized patients	Liberia	• Triage clinical emergencies in resource-limited settings (i.e. maternal sepsis, labor and delivery outside a clinical setting, and post-partum hemorrhage)
				• Appreciate maternal health disparities and cultural determinants of child-maternal health
	Disaster relief after a tsunami	Simulated patients	Indonesia	• Review the minimum standards for health systems in humanitarian relief settings
				• Develop clinical skills for the management of trauma in a disaster setting
Communicable diseases	Infant and adult diarrhea in a primary health care facility	Simulated patients	Haiti	• Identify public and personal/family sanitation measures for prevention of communicable diseases
				• Work in health care teams
	TB screening and management in urban primary health care facility	Standardized patients	Peru	• Study examples of public health policies for infection control
				• Develop patient communication methods for unexpected or poor patient outcomes
	Malaria eradication	Hypothetical exercise with student presentations	Namibia	• Understand the magnitude and common obstacles of public health campaigns
				• Apply evidence-based medicine in resource-limited areas
Non-communicable diseases	Hypertension screening and education in an urban community	Standardized patients	Ghana, Democratic Republic of Congo	• Develop effective communication and education tools based on health literacy level
				• Explore and understand traditional belief systems and social/cultural determinants of health
	Smoking cessation counseling	Standardized patients	China	• Understand the public health impact of tobacco use globally
				• Practice motivational interviewing
	Diabetes in an urban community	Standardized patients	Ecuador	• Demonstrate clinical empathy
				• Explore and respond appropriately to traditional healing methods and beliefs
Psycho-social illness	Physical and psychological trauma from torture	Standardized patients	Democratic Republic of Congo	• Appreciate the psychological, social, and physical impact of conflict and torture
				• Foster a safe and trusting healing environment
Language and communication	Working with interpreters	Standardized patients	NYC	• Develop skills that maximize communication using interpreters
				• Identify and address cross-cultural barriers to clinical communication

Prior to each session, students were required to complete background reading from the primary literature. The start of each session began with classroom discussion; the students were then given specific instructions and objectives for the simulation. Faculty members directly observed the encounters and debriefed with both students and actors after the simulation was complete, using an assessment tool developed to emphasize shared learning objectives across activities.

### Microbiology workshop

In addition to clinical simulations, students completed an interactive microbiology workshop on malaria parasitology. For this exercise, in three teams of 4 students each, students were given a hypothetical scenario in which they were given funding for malaria control/elimination in Namibia. In the scenario, the three teams each prepared a short presentation in one of three areas: rapid diagnostic tests, low-dose primaquine (with artemisinin combination therapy) for *P falciparum* gametocytocide, and vaccines. Faculty assigned students specific questions and journal articles from the primary literature to address during the discussion.

### Patient case discussions in tropical medicine

We designed 18 case discussions to enhance the students’ fund of knowledge on tropical diseases and global epidemiology. Under the guidance of a faculty member, each 60–90 min discussion was based on a real patient presentation (Table 4). Students prepared in advance using pathogen worksheets (Additional file 1: Supplement 1) covering selected infectious diseases on the differential diagnosis list. Discussions were led by students and facilitated by rotating faculty members.

**Table 4:** Selected case discussions

Case	Simulated site	Additional themes
Chagas disease	El Salvador	Global impact of neglected tropical diseases
Neurocysticercosis	Ecuador	Triaging altered mental status in resource-limited settings
Pulmonary tuberculosis in HIV/AIDS	Botswana	Migrant worker health; when to start HIV treatment
Asymptomatic HIV infection	Guinea	Female genital cutting; mother-to-child HIV transmission prophylaxis
Chronic hepatitis B virus infection and hepatocellular carcinoma	China	Global burden of cancer; traditional healing and herbal medicine
Neonatal tetanus	Viet Nam	Cultural determinants of health; vaccine-preventable diseases

### Journal club

In order to reinforce essential skills for evidence-based study and discussion, each student chose a scientific journal article with GH relevance to present to the class. The presentation consisted of a didactic exercise to inform the class of the clinical context, followed by classroom discussion to analyze the article’s evidence and impact on GH patient care and research.

### Conferences and lectures

Finally, students attended several weekly NYU conferences including ID case conference, ID grand rounds, and Pulmonary Medicine tuberculosis case conference. NYU and visiting faculty with GH expertise provided lectures covering a variety of topics, including global burden disease, health systems, telemedicine, vaccine programs and disease eradication, child health metrics, and careers in GH.

### Assessment and evaluation

Assessment and evaluation are terms that both play important and distinct roles in curricular development. Here, assessment refers to measurement of student performance, and evaluation refers to measurement of the curriculum itself.

For this initial 3-year pilot phase of this elective clerkship, assessment of student performance relied primarily on direct faculty observation and feedback. For each activity, we applied shared GH learning objectives (Table 1). For the clinical skills simulations, faculty debriefed with the student and the actor using a student simulation assessment tool checklist (Additional file 1: Supplement 2), with student-to-faculty direct observation ratios ranging from 6 students per faculty member to 1 student per faculty member. We also provided students direct detailed written feedback for journal club presentations and clinical write-ups. For the case discussions, faculty used a student case discussion assessment tool to measure performance (Additional file 1: Supplement 3).

To evaluate the curriculum, students received electronic surveys upon completion of the block, to evaluate how well each activity met the shared learning objectives throughout the course. Each year, evaluations were expanded and revised according to our growing experience with the selective, and each year the evaluations became more competency-based. All evaluations were distributed and reviewed by the NYU Institute for Innovations of Medical Education (IIME). All data presented here are anonymous and blinded to the authors by IIME, and compliant with IIME’s active Institutional Review Board approval for curricular evaluation.

For the initial 2012 cohort evaluation, curricular activities were grouped into topic categories, and students were asked to rate the improvement in their knowledge of clinical topics after each activity: 1) same knowledge, 2) a little more, or 3) much more. In addition to topics, clinical skills were rated in this same way, including patient communication, self-directed learning, evidence-based review, peer to peer learning, public health campaigns, interdisciplinary health teams, and ethics, human rights and patient advocacy. Three students were excluded because they enrolled outside of the concentration system.

All 2013 students were asked to identify ways the elective influenced their career interests, goals, and overall clinical skills. This cohort was excluded from quantitative measures, due to unforeseen delays related to the closure of NYU School of Medicine facilities from Hurricane Sandy in New York City in 2012 and 2013.

All 2014 students were asked to evaluate the degree to which the elective contributed to their abilities to fulfill each of the shared learning objectives listed in Table 1. In this way, each year expanded and adapted the evaluation tools according to shared GH competencies. Overall, 18 of 33 students were included to receive quantitative-qualitative surveys upon completion of the course.

## Results

**Fig. 1:**
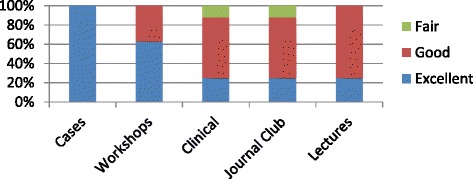
Student feedback: individual course components (4-point Likert scale, Poor-Fair-Good-Excellent)

Over the course of this 3-year pilot, student and faculty feedback for the elective was consistently positive. The response rate for quantitative surveys was 78 %. Eighty-six percent rated the elective as excellent. Among each activity, case discussions and experiential learning simulations (workshops) received the highest ratings (Fig. 1).

**Fig. 2:**
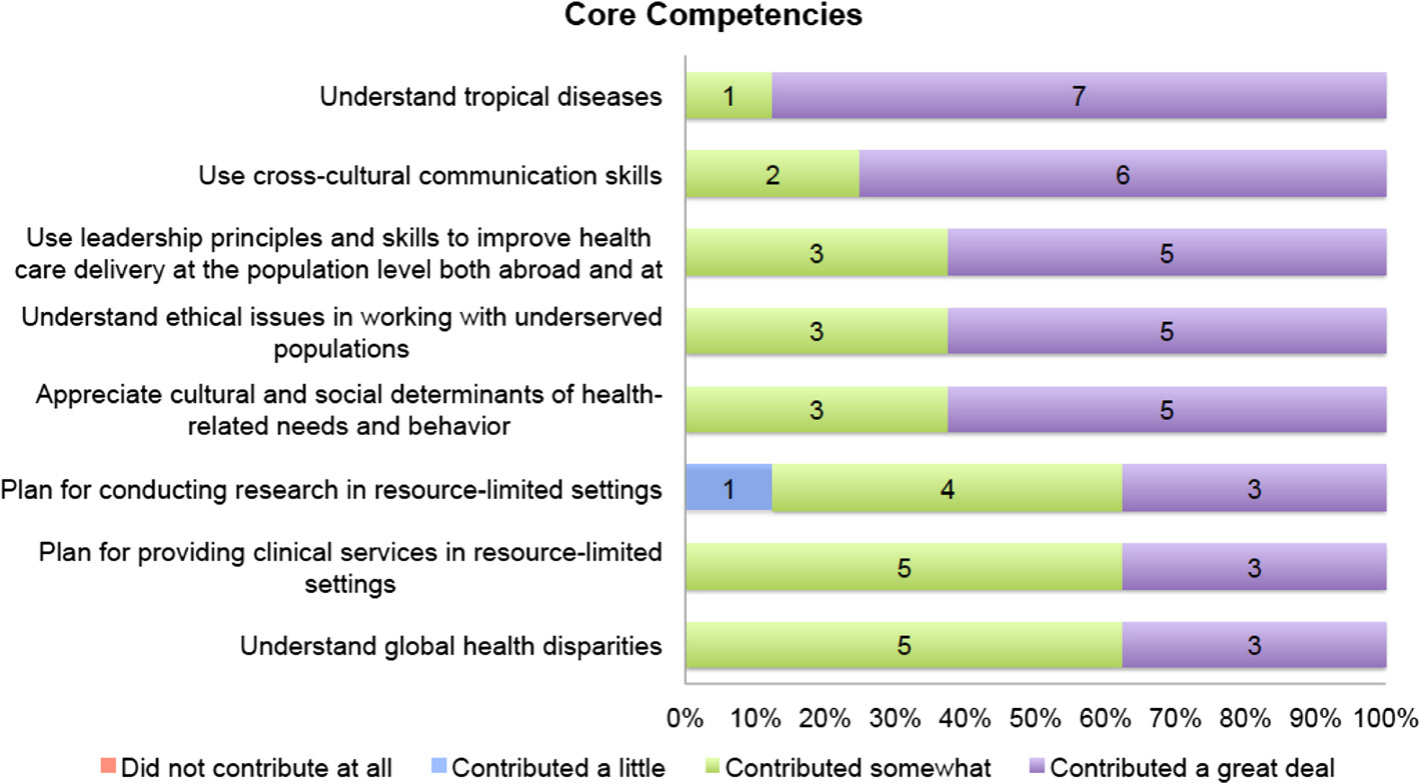
Student evaluation of core competencies. Core Competencies. To what degree did the selective contribute to your ability to fulfill each of the following core competencies in Global Health:

When measuring core competencies, the elective provided the greatest contribution to students’ knowledge of tropical diseases and skills in cross-cultural communication, followed by leadership principles to improve health care delivery, understanding of ethical issues in working with underserved populations, and appreciation of cultural and social determinants of health-related needs and behavior (Fig. 2).

Qualitative evaluation by students was also consistently positive (Table 5).

**Table 5:** Selected feedback by students

2012	• [GH is] really important for any physician who wants to think outside the boundaries of this country.
	• [At Hansen’s disease clinic at BHC], we saw a variety of patients from all over the world.
	• Fantastic hands-on training that really took us out of the classroom and forced us to think on our feet, which is so different from sitting at a desk with a book. A real introduction to the transition from studentship to practitioner.
	• With case discussions, I really solidified my ability to evaluate a patient completely.
2013	• A year after completing the GH elective, it helped me gain more cultural competence to communicate across cultural barriers, and to improve my knowledge of infectious diseases.
	• My career goals were impacted to understand and serve in resource-limited settings both in the USA and abroad.
2014	• Strengths included individual feedback, becoming more comfortable in the simulation center, and learning about various tropical diseases.
	• This was one of my favorite months in medical school with a great variety of activities.
	• One suggestion is to ask students to pick a country and research it, and present or write-up the profile of that country.

For each offering, at least thirty faculty members and fellows volunteered, yielding over 215 direct contact teaching hours each year and representation from 11 departments (Table 6).

**Table 6:** Number of faculty and fellows from participating Departments at NYUSOM

Department/Division	Spring 2012	Spring 2013	Spring 2014
Medicine/Division of Gen Internal Medicine	3	3	6
Division of Infectious Diseases	13	14	14
Division of Pulmonary Medicine	2	2	2
Division of Gastroenterology	1	1	1
Pediatrics	2	5	4
Pathology	1	1	1
Microbiology	3	3	2
Population Health	3	3	5
Psychiatry	4	4	3
Emergency Medicine	2	2	2
Obstetrics & Gynecology	2	2	3
Surgery	2	1	1
Radiology	1	1	1
Dermatology	1	1	2
Total faculty	40	43	48

## Discussion

After three years, the initial pilot phase of the GH Selective exceeded expectations. Teaching activities improved and expanded with each offering, GH competency-based assessments were increasingly utilized, and student feedback was overwhelmingly positive. Moving forward, the selective is now offered twice annually as a prerequisite to a GH concentration at NYUSOM.

Clinical case discussions and experiential learning through simulated exercises were found to be the greatest strengths of the selective. Simulation education and standardized patients have taken a unique and central role in medical education [19], including training to work with cross-cultural populations [20]. While medical students at NYU have had the opportunity to participate in fieldwork overseas for many years, this selective offered them a controlled setting on campus to gain and practice essential skills prior to an overseas rotation. The selective also provided students a structured immersion in local health disparities, underlining the point that global health is local health first. Additionally, embedded within each of these activities was an emphasis on cultural sensitivity training, a notable priority for medical curricula on a national level. Faculty facilitators discussed communication skills, health literacy, health navigation, and ethics in each component activity. Key learning objectives in motivational interviewing, working with adult learners, triaging priorities, and enhancing empathy and trust in the patient encounter were also included.

An additional strength was seen in the scope of faculty participation. A remarkably high degree of interest and commitment to the topic across all disciplines at our institution was shown, which highlights a diverse spectrum of expertise at a major academic health center. The exceptionally high number of departments involved indicates that global health training can and should draw across the full spectrum of medical education, and to extend to include other health professions.

Both a strength and a challenge, the intensive faculty time involved was a limitation for offering this course more frequently. To that end, simulation can serve two very important purposes: in addition to providing experiential learning opportunities, the standardized patient offers a reliable resource for standardized objective structured clinical assessment of student competencies. Specifically, improved assessment measures in the form of standardized checklists will allow the course’s activities to be scaled up, disseminated and adapted to different learners with greater success, while at the same time integrating and mapping key cultural competency objectives that build on similar themes in the larger medical student curriculum.

## Conclusions

With a special emphasis on cross-cultural sensitivity, this pilot selective trained future practitioners in fund of knowledge, clinical skills, and service delivery methods relevant to both global and to culturally-diverse domestic settings. Similar to other GH education programs, this pilot informed students about future choices for ethical and appropriate training and career options in GH [21], with the goal to avoid counterproductive GH efforts that cause more harm than good. Ultimately, this pilot course demonstrated that global health transcends any single department, discipline, disparity, or region, and serves our institution’s core missions in education, patient care, and research.

## Additional file

Additional file 1: Supplement 1. Pathogen worksheet (Case Study Guide). Supplement 2: Student assessment tool for clinical skills simulations. Supplement 3: Student assessment tool for case discussions.

## Abbreviations

GH: global health
AAMC: American Association of Medical Colleges
NYU: New York University
C21: Curriculum for the 21^st^ Century
BHC: Bellevue Hospital Center
ID: Infectious Diseases
NYSIM: New York Simulation Center for the Health Sciences, Institute for Innovations in Medical Education
IIME: Institute for Innovations in Medical Education
