# Supplementary material for: Teaching global health with simulations and case discussions in a medical student selective
Source: Global Health. 2015 Jul 4;11:28. doi: 10.1186/s12992-015-0111-2 (PMC4491235; doi:10.1186/s12992-015-0111-2)
Supplement: Additional file 1: Supplement 1. — Pathogen worksheet (Case Study Guide). Supplement 2: Student assessment tool for clinical skills simulations. Supplement 3: Student assessment tool for case discussions. [file 12992_2015_111_MOESM1_ESM.docx]

**Supplement 1: Pathogen worksheet (Case Study Guide)**

*A self study “checklist” tool to prepare for Case Discussions*

Use the *Oxford Handbook of Tropical Medicine*, web links on ALEX, and library digital resources such as *Up-To-Date* and *Harrison’s Principles of Internal Medicine*.

One can “fill-in-the-blanks” for each criterion below while studying, during Independent study time in order to prepare for the group case discussions.

**Students are welcome and encouraged to have these notes in-hand during classroom discussions, in addition to pocket guides like the Oxford Handbook. A blank template is on the second page.**

**Pathogen**

Nature: morphology, relevant physiology: Medical importance varies with particular pathogens. Morphology of the malaria parasite, other protozoa, some helminths and a few bacteria is important. Importance of pathogen physiology also varies; ex: knowing a pathogen is an anaerobe is important; knowing pathogen nutrient requirements can also be important

Life cycle: Route of transmission, zoonotic aspects, important life cycle stages, points in cycle to disrupt transmission by medical and/or public health measures

**Disease**

Risk factors: Geographic, environmental *[regional, season/weather, sanitation]*, life styles *[occupation, personal habits, exposure to water with pathogens, housing, access to care/prevention]*, genetic/idiosyncratic susceptibilities

Common presentations and range of presentation

Pathogenesis: Mechanisms, virulence factors, organs affected, range of outcomes if treated/untreated

Factors that affect prognosis: Patient age, inoculum size, genetic/idiosyncratic factors, immune status, co-infections, residuals/sequelae

Medical emergencies: Situations/conditions under which infection can present as a medical emergency

**Diagnosis**

Signs and symptoms

Significant history

Physical

Differential diagnosis

Laboratory studies: Principle, sensitivity/specificity, availability/cost, appropriateness for developing/developed countries

**Treatment**

Anti-infectives: Efficacy, PD, PK, ADR, ease of administration/compliance, availability/cost, appropriateness for developing/developed countries, special consideration

Supportive treatment: Including appropriateness for developing/developed countries

Confounding issues:

**Traveler care**

Pre-travel exam

Pre-travel Interventions: Chemoprophylaxis, vaccines

Pre-travel advice: Food/water, vector avoidance, signs/symptoms indicating need to seek medical care

Post-travel exam

Post travel issues: Treatment of diagnosed disease, prognosis, monitoring

**CASE STUDY GUIDE:**

**DISEASE:**

**Pathogen**

Nature:

Life cycle:

Vector:

**Disease**

Risk factors:

Common presentations and range of presentation:

Pathogenesis:

Factors that affect prognosis:

Medical emergencies:

**Diagnosis**

Signs and symptoms:

Significant history:

Physical:

Differential:

Laboratory studies:

**Treatment**

Anti-infectives:

Supportive treatment:

Confounding issues:

**Traveler care**

Pre-travel exam

Pre-travel Interventions:

Pre-travel advice:

Post-travel exam:

Post travel issues:

**Supplement 2: Student assessment tool for clinical skills simulations**

Student:

Simulation:

Faculty:

| **Skill** | **Objective** | **Not done** | **Partially done** | **Well done** | **Comments** |
| --- | --- | --- | --- | --- | --- |
| Information gathering | - Posed one question at a time - Used open-ended questions - Repeated information for clarity - Elicited pertinent symptomatology and history |  |  |  |  |
| Relationship development | - Improved rapport with non-verbal behavior (eye contact, posture) - Responded appropriately to patient’s emotions - Used easily-understood words |  |  |  |  |
| Practice-based learning | - Recognized and adapted to resource-limited settings - Created cost-effective, evidence-based, patient-centered treatment plans |  |  |  |  |
| Education and counseling | - Checked for patient’s understanding and assessed health literacy - Explained clearly - Used the Teach-Back method - Addressed patient’s concerns and questions appropriately |  |  |  |  |
| Cultural sensitivity | - Employed the BATHE technique for clinical empathy - Explored cultural and social determinants of heath - Did not dismiss or judge patient’s belief systems - Addressed barriers to communication and to healthy behaviors |  |  |  |  |
| Professionalism | - Used respectful tone - Was sensitive/responsive to patients needs - Handled own emotions appropriately - Worked well within the team - Demonstrated understanding of ethical issues related to patient needs |  |  |  |  |

**Supplement 3: Student assessment tool for case discussions**

Student:

Case:

Faculty:

|  | **Excellent** | **Good** | **Average ** | **Below Average ** | **Comments** |
| --- | --- | --- | --- | --- | --- |
| **Preparation**:  Demonstrates knowledge of:  Portal of entry  Risk factors  Pathogenesis  Natural history |  |  |  |  |  |
| **Information gathering**:  Elicits salient  information from  case history  Makes reasonable interpretations  Suggests when additional information is needed |  |  |  |  |  |
| **Management**:  Evaluates disease  severity and  intervention urgency  Knows, understands, and is able to discuss  therapy options  Develops reasonable management plan Predicts probable outcomes |  |  |  |  |  |
| **Professionalism**:  Respectful  Works well within the group  Offers teaching points based on  student’s own expertise |  |  |  |  |  |
